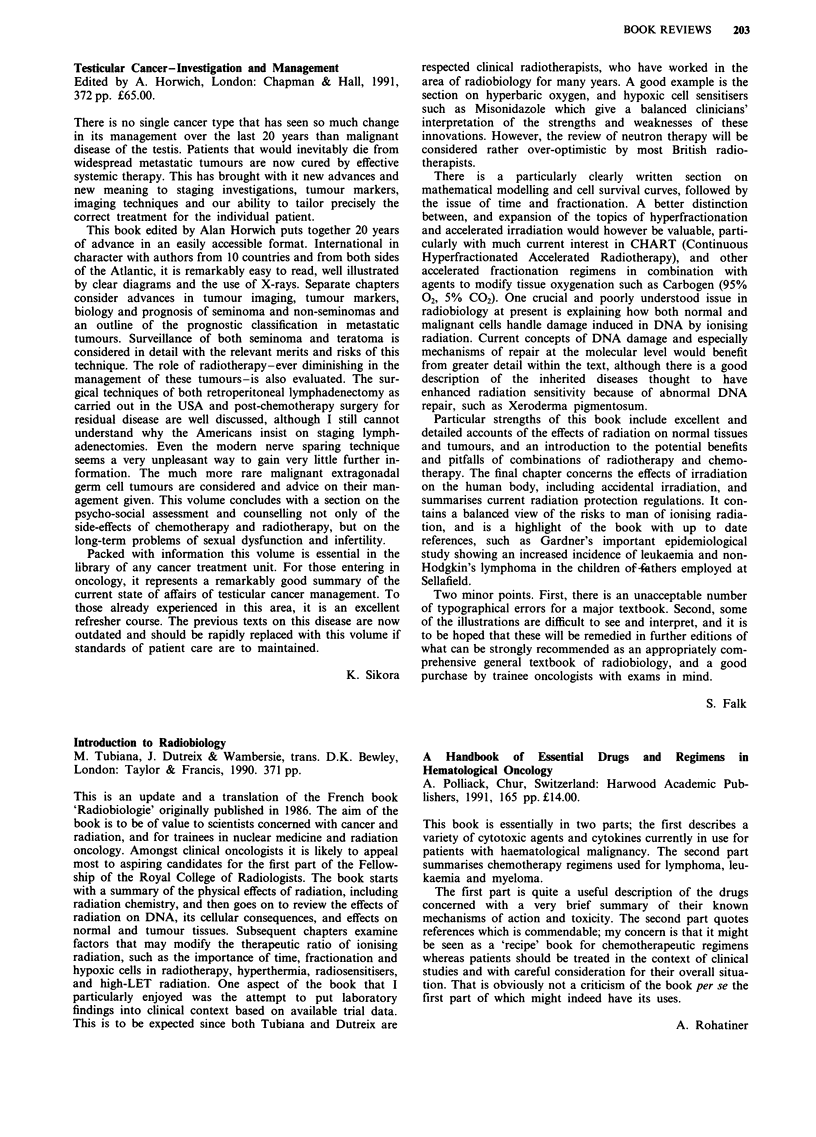# Introduction to Radiobiology

**Published:** 1993-01

**Authors:** S. Falk


**Introduction to Radiobiology**
M. Tubiana, J. Dutreix & Wambersie, trans. D.K. Bewley, London: Taylor & Francis, 1990. 371 pp.

This is an update and a translation of the French book 'Radiobiologie' originally published in 1986. The aim of the book is to be of value to scientists concerned with cancer and radiation, and for trainees in nuclear medicine and radiation oncology. Amongst clinical oncologists it is likely to appeal most to aspiring candidates for the first part of the Fellowship of the Royal College of Radiologists. The book starts with a summary of the physical effects of radiation, including radiation chemistry, and then goes on to review the effects of radiation on DNA, its cellular consequences, and effects on normal and tumour tissues. Subsequent chapters examine factors that may modify the therapeutic ratio of ionising radiation, such as the importance of time, fractionation and hypoxic cells in radiotherapy, hyperthermia, radiosensitisers, and high-LET radiation. One aspect of the book that I particularly enjoyed was the attempt to put laboratory findings into clinical context based on available trial data. This is to be expected since both Tubiana and Dutreix are respected clinical radiotherapists, who have worked in the area of radiobiology for many years. A good example is the section on hyperbaric oxygen, and hypoxic cell sensitisers such as Misonidazole which give a balanced clinicians' interpretation of the strengths and weaknesses of these innovations. However, the review of neutron therapy will be considered rather over-optimistic by most British radiotherapists.

There is a particularly clearly written section on mathematical modelling and cell survival curves, followed by the issue of time and fractionation. A better distinction between, and expansion of the topics of hyperfractionation and accelerated irradiation would however be valuable, particularly with much current interest in CHART (Continuous Hyperfractionated Accelerated Radiotherapy), and other accelerated fractionation regimens in combination with agents to modify tissue oxygenation such as Carbogen (95% $O_2$, 5% $CO_2$). One crucial and poorly understood issue in radiobiology at present is explaining how both normal and malignant cells handle damage induced in DNA by ionising radiation. Current concepts of DNA damage and especially mechanisms of repair at the molecular level would benefit from greater detail within the text, although there is a good description of the inherited diseases thought to have enhanced radiation sensitivity because of abnormal DNA repair, such as Xeroderma pigmentosum.

Particular strengths of this book include excellent and detailed accounts of the effects of radiation on normal tissues and tumours, and an introduction to the potential benefits and pitfalls of combinations of radiotherapy and chemotherapy. The final chapter concerns the effects of irradiation on the human body, including accidental irradiation, and summarises current radiation protection regulations. It contains a balanced view of the risks to man of ionising radiation, and is a highlight of the book with up to date references, such as Gardner's important epidemiological study showing an increased incidence of leukaemia and non-Hodgkin's lymphoma in the children of fathers employed at Sellafield.

Two minor points. First, there is an unacceptable number of typographical errors for a major textbook. Second, some of the illustrations are difficult to see and interpret, and it is to be hoped that these will be remedied in further editions of what can be strongly recommended as an appropriately comprehensive general textbook of radiobiology, and a good purchase by trainee oncologists with exams in mind.

S. Falk